# Identification of Silent Myocardial Ischemia in Patients with Long-Term Type 1 and Type 2 Diabetes

**DOI:** 10.3390/ijerph19031420

**Published:** 2022-01-27

**Authors:** Dominika Rokicka, Anna Bożek, Marta Wróbel, Alicja Nowowiejska-Wiewióra, Aleksandra Szymborska-Kajanek, Tomasz Stołtny, Mariusz Gąsior, Krzysztof Strojek

**Affiliations:** 1 Department of Internal Medicine, Diabetology and Cardiometabolic Disorders, Faculty of Medical Sciences Zabrze, Medical University of Silesia, 41-800 Zabrze, Poland; mwrobel@sum.edu.pl (M.W.); kstrojek@sum.edu.pl (K.S.); 2 Outpatient Clinic for Patients with Diabetes, 41-800 Zabrze, Poland; anna_bozek@poczta.fm (A.B.); olutkas@wp.pl (A.S.-K.); 3 3rd Department of Cardiology, Faculty of Medical Sciences Zabrze, Medical University of Silesia, 41-800 Zabrze, Poland; anwiewiora@tlen.pl (A.N.-W.); mgasior@sum.edu.pl (M.G.); 4 District Hospital of Orthopaedics and Trauma Surgery in Piekary Śląskie, 41-940 Piekary Śląskie, Poland; quattro42@poczta.onet.pl

**Keywords:** type 1 diabetes, type 2 diabetes, silent myocardial ischemia

## Abstract

(1) Background: This study aimed to analyze epidemiological data to identify risk factors for silent myocardial ischemia in patients with long-term type 1 and type 2 diabetes. (2) Methods: An analysis was performed on 104 patients with long-term type 1 and type 2 diabetes who had not previously been diagnosed with cardiovascular disease. During hospitalization, patients were subjected to a standard ECG exercise test on a treadmill. If the test could not be performed or the result was uncertain, a pharmacological exercise test with dobutamine was performed. In the case of a positive exercise ECG test or a positive dobutamine test, the patient underwent coronary angiography. (3) Results: Atherosclerotic lesions were found in 24 patients. Patients with silent ischemia were significantly older and had a lower mean left ventricular ejection fraction and a higher incidence of carotid atherosclerosis. The presence of microvascular complications did not increase the risk of silent ischemia. (4) Conclusions: Silent heart ischemia is more common in type 2 than type 1 diabetes. Predisposing factors include older age, coexistence of carotid atherosclerosis, lower left ventricular ejection fraction, and smoking in patients with type 1 diabetes. Concomitant microvascular complications are not a risk factor.

## 1. Introduction

Cardiovascular complications are the leading cause of death in populations with diabetes. A significant problem in this group of patients is the form of ischemic heart disease, which in the absence of clinical signs, does not allow for the implementation of appropriate diagnostic and therapeutic procedures to reduce the risk of acute coronary syndrome. In 1999, Janand-Delenne et al. observed that every fifth patient with diabetes had a critical stenosis, as evidenced by an angiography of the coronary arteries. This was despite a negative history of stenocardial pain or other features of the ischemic disease [1]. In recent years, significant progress in the treatment of diabetes has been made, which has resulted in a reduction in the incidence of micro- and macroangiopathic complications [2,3]. This study aimed to verify epidemiological data from several years and to analyze risk factors, based on clinical and laboratory data, which increased the risk of coronary heart disease.

## 2. Material and Methods

### 2.1. Material

The analysis included 104 patients with diabetes hospitalized in 2013–2017 in the Department of Internal Medicine, Diabetology, and Cardiometabolic Disorders in Zabrze to perform standard diagnostics for ischemic heart disease due to high or very high risk of cardiovascular diseases, without clinical symptoms and prior diagnosis of cardiovascular disease.

Inclusion criteria:

- Type 1 diabetes for at least 10 years;
- Type 2 diabetes for at least 5 years.

Exclusion criteria:

- Diagnosed cardiovascular complications (chronic and acute coronary syndromes, stroke, and lower limb atherosclerosis);
- Features of ischemic heart disease in physical examination (specific and non-specific symptoms) and/or in ECG.

The patients were informed in detail about the purpose of the study and the method of its conduct. Permission to conduct the study was obtained from all participants. The study was approved by the Bioethics Committee at the Medical University of Silesia in Katowice.

### 2.2. Methods

Silent ischemia was defined based on positive results from the treadmill exercise test or the dobutamine test in the absence of angina pain or its equivalents. Silent ischemia was not diagnosed in patients who were not qualified for coronary angiography after negative results of the treadmill exercise or dobutamine test.

At the time of admission to the hospital, the following data were collected from patients:

(1) History—age, gender, duration and method of diabetes treatment, presence of arterial hypertension, smoking, alcohol, presence of microvascular complications (nephropathy, neuropathy, and retinopathy), and family history of ischemic heart disease;
(2) Anthropometric measurements;
(3) Venous blood samples taken for laboratory tests, including HbA1c, total cholesterol, LDL, HDL, triglycerides, hsCRP, NT-proBNP, creatinine, and eGFR;
(4) Echocardiographic examination;
(5) Doppler ultrasound of carotid arteries;
(6) The treadmill stress test performed according to Bruce’s protocol;
(7) Twenty-four-hour Holter ECG examination;
(8) The dobutamine test (in patients with undiagnostic or questionable results of the treadmill exercise test).

In the case of a positive stress test result, patients were qualified for coronary angiography. If the test was impossible to perform or the result was doubtful, a pharmacological exercise test with dobutamine was performed—a positive result was an indication for coronary angiography. In the case of a negative exercise test result or a normal dobutamine test, the diagnosis for ischemic heart disease was terminated and its presence was not confirmed.

In patients qualified for coronary angiography, silent myocardial ischemia was diagnosed. Based on the performed coronary angiography, the presence of hemodynamically significant or insignificant atherosclerotic lesions in the coronary arteries was assessed.

## 3. Statistical Analysis

The analysis of the results was performed using MedCalc Statistical Software v. 14.12.0 (MedCalc Software bvba, version 19.7, Ostend, Belgium; http://www.medcalc.org; 2014 (accessed on 25 February 2021)). Data imputation was not used. Continuous variables are presented as mean ± standard deviation in the case of a normal distribution. The distribution of results was assessed with the Shapiro–Wilk test.

When comparing the distributions of continuous variables in patients with diabetes type 1 and 2, the chi-squared test (qualitative variables) and the ANOVA test (continuous variables) were used.

Based on logistic regression, the odds ratio was calculated for the occurrence of asymptomatic coronary artery disease in relation to the factors mentioned in the data analysis. The statistically significant factors were included in the multiple logistic regression models (including all factors and backward regression).

## 4. Results

A total of 104 patients participated in the study, including 37 patients with type 2 diabetes (35.6%) and 67 patients with type 1 diabetes (64.4%). All patients underwent an exercise test according to the Bruce protocol.

The general clinical characteristics of the study group are presented in Table 1.

**Figure 1:**
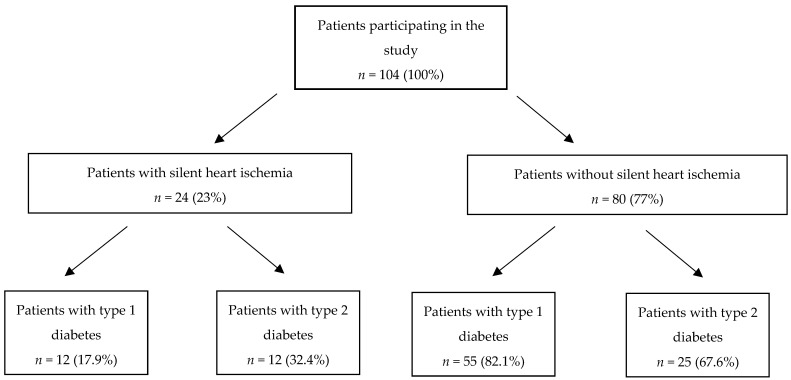
Detailed distribution of patients with or without silent ischemia.

In all patients, the exercise treadmill test was clinically negative (no stenocardial symptoms), while the electrocardiography was as follows:

(a) Positive in 18 patients (7 with type 1 diabetes and 11 with type 2 diabetes);
(b) Questionable in 11 patients with type 1 diabetes;
(c) Undiagnostic in 5 patients (2 with type 1 diabetes and 3 with type 2 diabetes);
(d) Negative in 70 patients (47 with type 1 diabetes and 23 with type 2 diabetes) (Figure 1).

The dobutamine test was performed in 15 patients with doubtful or non-diagnostic results of the exercise test. One patient who qualified for the dobutamine test, according to the above criteria, did not consent to the test. In this case, a coronary angiography was performed, omitting the stage of the echocardiographic stress test. A positive dobutamine test result was obtained in 5 patients (4 with type 1 diabetes and 1 with type 2 diabetes) and negative in 10 patients (8 with type 1 diabetes and 2 with type 2 diabetes).

A coronary angiography was performed in a group of 24 patients who qualified for the study based on a positive exercise test result or a positive dobutamine test result. Detailed distribution of patients with or without silent ischemia are presented in Figure 1.

The presence of atherosclerotic lesions was found in 20 patients (19%) (9 with type 1 diabetes and 11 with type 2 diabetes). Significant hemodynamic changes were visualized in 8 patients, and hemodynamically insignificant changes were found in 16 patients.

In 4 patients (3 with type 1 diabetes and 1 with type 2 diabetes), coronary artery disease was excluded based on the coronary angiography. In these patients, silent ischemia due to microcirculation dysfunction was diagnosed.

None of the patients had abnormalities in the 24 h Holter ECG examination that could indicate myocardial ischemia.

The factors that could potentially influence the occurrence of silent myocardial ischemia were analyzed. The mean age of patients with silent ischemia was statistically and significantly higher compared to the group without myocardial ischemia (58.0 ± 11.6 vs. 45.0 ± 14.1 years, respectively; *p* < 0.001). The group of patients with silent ischemia had a lower mean, left ventricular ejection fraction (53.9 ± 3.7% vs. 56.5 ± 3.2%, respectively; *p* = 0.001) and a higher incidence of carotid atherosclerosis (75% vs. 41%, respectively, 3% of patients; *p* = 0.004).

Patients with silent ischemia with type 2 diabetes were older than patients with silent ischemia with type 1 diabetes (64.0 ± 10.0 vs. 52.0 ± 10.1 years; *p* = 0.01) and had a significantly shorter duration of diabetes (15.2 ± 6.6 vs. 27.0 ± 11.1 years). Nonischemic patients with type 1 diabetes were younger, but they manifested the longest diabetes duration (Table 2).

Comparing the entire groups of patients with type 1 and type 2 diabetes, a significantly higher incidence of microvascular complications in the form of diabetic retinopathy (*p* = 0.004) and sensorimotor polyneuropathy (*p* = 0.03) was observed in the group of patients with type 1 diabetes. The presence of microvascular complications did not increase the risk of silent ischemia. Patients with type 1 diabetes had a better lipid profile, significantly higher levels of HDL cholesterol (1.8 ± 0.44 mmol/L vs. 1.4 ± 0.56 mmol/L, respectively; *p* < 0.001), and lower triglyceride concentrations (1.1 ± 0.59 mmol/L vs. 1.8 ± 1.29 mmol/L, respectively; *p* < 0.001) than patients with type 2 diabetes. There were no differences in total cholesterol, LDL cholesterol, non-HDL cholesterol, creatinine, NT-proBNP, and hsCRP among the groups.

Table 3 presents the results of multiple logistic regression for the factors contributing to the occurrence of silent ischemia. There was a significant influence of age and a presence of carotid atherosclerosis on the occurrence of silent heart ischemia. Additionally, with each 1% decrease in the mean left ventricular ejection fraction (EF), the risk of ischemia was 27% higher (*p* < 0.01). All abovementioned parameters were significant regarding both types of diabetes together and for patients with type 1 diabetes. In patients with type 1 diabetes, a significant influence of smoking was also present (the risk of ischemia is 400% higher (*p* < 0.05).

For each decade of life, the risk of ischemia in patients with diabetes increased by 106% (*p* < 0.001) and in patients with type 1 diabetes increased by 153% (*p* < 0.01).

### Safety

No side effects were recorded during the study.

## 5. Discussion

The present study shows that long-term type 1 and type 2 diabetes increase the risk of silent myocardial ischemia. It is much more common in patients with type 2 diabetes (32.4%) than in patients with type 1 diabetes (17.9%). Factors predisposing patients to silent myocardial ischemia are older age; the coexistence of carotid atherosclerosis; lower left ventricular ejection fraction; and in patients with type 1 diabetes, smoking.

In times of great progress in the treatment of diabetes, the introduction of new hypoglycemic drugs and better methods of glycemic self-control enable the intensification of hypoglycemic treatment and the achievement of target glucose levels. Many patients still suffer from macrovascular and microvascular complications of diabetes.

Cardiovascular complications are responsible for over 75% of deaths in patients with diabetes [4,5]. They are much more common in patients with long-term type 2 diabetes than in patients with type 1 diabetes. This also applies to silent myocardial ischemia, which was confirmed in our study.

The available literature data indicate that the assessment of the presence of silent myocardial ischemia in patients with diabetes is often limited to the study of one group of patients. That is, it concerns patients with either type 2 diabetes or type 1 diabetes. Our study included patients with both types of diabetes. This provided the opportunity to compare these two groups of patients who underwent the same diagnostic tests. In 1999, Janand-Delenne B. et al. assessed the incidence and risk factors of silent myocardial ischemia in a population of patients with type 2 diabetes and patients with type 1 diabetes [1]. In this study, 20.9% of men with type 2 diabetes and only 4.2% of patients with type 1 diabetes were diagnosed with silent myocardial ischemia. The results for patients with type 2 and type 1 diabetes do not match our data. In both studies, the age of the patients, disease duration, and BMI were comparable.

The differences were in the number of patients analyzed. In the study by Janand-Delenne B. et al., most of the patients were diagnosed with type 2 diabetes and only 35% of the study group were patients with type 1 diabetes. In our study, 64% were patients with type 1 diabetes [1]. Prasad et al. estimated the incidence of silent heart ischemia at 23.1% in the group with type 2 diabetes [6]. In a study by Chico A. et al., the incidence of silent ischemia in the entire study group (including patients with type 1 and type 2 diabetes) confirmed by angiography was 3.7% [7].

Differences in the incidence of silent ischemia in patients with diabetes across studies are most likely due to the use of different diagnostic screening tests. Until recently, according to the cardiological guidelines for screening, exercise testing was the primary method of assessing the risk of silent heart ischemia.

Currently, the sensitivity and specificity of the exercise ECG test in the population with diabetes have been estimated at 47% and 81%, respectively [8]. According to the latest recommendations, a resting ECG is recommended in patients with diabetes and diagnosed arterial hypertension or with suspected coronary artery disease [9,10]. Assessments of the presence of atherosclerotic plaques in the ultrasound of the carotid and femoral arteries, and coronary calcification using computed tomography (CT), should be considered to assess the risk of coronary artery disease in asymptomatic patients with diabetes and moderate risk of cardiovascular disease [10]. Coronary CT angiography or functional imaging (radioisotope imaging of myocardial perfusion, stress cardiac magnetic resonance, or exercise echocardiography with pharmacological stress) may be considered in asymptomatic patients with diabetes as part of screening for coronary disease [10,11,12,13]. In our study, we used both tests, the stress ECG test and the dobutamine stress echocardiography. We assessed the presence of atherosclerotic plaques in the ultrasound of the carotid arteries and confirmed the presence of atherosclerotic lesions in the coronary arteries by performing coronary angiography.

In addition to the epidemiological assessment, risk factors for cardiovascular disease in patients with diabetes and silent ischemia were assessed. The age of the patient, the coexistence of a lower left ventricular ejection fraction, and the presence of atherosclerotic lesions in the carotid arteries had a decisive influence on the occurrence of silent ischemia.

The relationship between diabetes and congestive heart failure (CHF) as well as its impact on the prognosis in patients with diabetes is well known and has been extensively documented. In many studies, patients with diabetes had a lower left ventricular ejection fraction (LVEF) than those without diabetes. The higher incidence of coronary artery disease in patients with diabetes was indicated as the cause of the lower LVEF [14,15,16]. However, large epidemiological studies confirmed that diabetes was an independent risk factor for the development of heart failure. The lower left ventricular ejection fraction in patients with diabetes was not associated with the presence of arterial hypertension, coronary artery disease, or valvular disease [17,18]. Thus, in patients with diabetes, other pathogenetic mechanisms lead to left ventricular systolic dysfunction. Perhaps, as suggested by Witteles and Fowler, insulin resistance is the main etiological factor in the development of heart failure in patients with diabetes [19]. Thus, a reduced left ventricular ejection fraction in a patient with diabetes may be a risk factor for the development of cardiovascular disease (including silent heart ischemia) rather than a consequence of its occurrence.

Many researchers, while assessing the risk factors of cardiovascular disease in patients with type 2 diabetes, emphasized the importance of screening for carotid atherosclerosis [20,21]. Currently, the presence of atherosclerotic plaques, and not the evaluation of the intima-media complex in the ultrasound of carotid arteries, is a factor modifying the cardiovascular risk in asymptomatic patients with diabetes [10].

Smoking is a strong independent risk factor for the development of cardiovascular disease. Due to damage to the vascular endothelium, a reduction in nitric oxide production, an overproduction of pro-clotting factors in blood vessels, and changes in plasma lipoproteins, atherosclerotic changes in arteries are intensified [22]. It seems that the cardiovascular risk should be the same in smoking patients with diabetes, regardless of the type of diabetes. In our study, we observed that smoking increased the incidence of silent ischemia in patients with type 1 diabetes. This relationship was not observed in patients with type 2 diabetes.

Observing other diabetic complications in a meta-analysis, Xiaoling Cai et al. found that, in patients with type 1 diabetes who smoked, the risk of developing diabetic retinopathy increased significantly, with a significant decrease in the incidence of this complication in smokers with type 2 diabetes. However, these reports should not affect the current recommendations on the need to quit smoking in all patients with diabetes [23].

The presence of microangiopathic complications and the features of dyslipidemia are recognized risk factors for coronary artery disease. The results of our study do not confirm this. We found that the higher incidence of microvascular complications, in the form of diabetic retinopathy and sensorimotor polyneuropathy in the group of patients with type 1 diabetes, was not associated with an increased risk of silent ischemia. This is consistent with the results obtained by Llaurado et al., where patients with type 1 diabetes were assessed [24].

In our study, patients with type 1 diabetes were characterized by a better lipid profile compared to patients with type 2 diabetes, by significantly higher levels of HDL cholesterol, and by lower levels of triglycerides. Worse lipid profile in patients with type 2 diabetes in our study was associated with a higher incidence of silent ischemia (Figure 1)., These data are consistent with the risk assessment of coronary artery disease by Lee J.S. et al. In this study, patients with diabetes and high triglycerides, combined with low HDL cholesterol levels, had increased risk of coronary artery disease and ischemic stroke [25]. In the study by Ran J.S. et al., the population with diabetes and LDL cholesterol <100 mg/dL and high triglycerides and low HDL cholesterol had an estimated 35–62% higher risk of coronary heart disease in the 10-year follow-up compared to those with normal triglyceride levels and HDL cholesterol [26].

Due to the wide variety of diagnostic methods used to assess silent heart ischemia in studies on patients with diabetes, often assessing only one group of patients with type 2 or type 1 diabetes, it seems necessary to conduct further studies to develop effective and standardized screening tests and to assess risk factors for cardiovascular disease.

## 6. Conclusions

(1) The prevalence of silent myocardial ischemia in long-term diabetes is high.
(2) Silent ischemia of the heart is much more common in patients with type 2 diabetes than in patients with type 1 diabetes.
(3) The factors that predispose patients to silent myocardial ischemia include
  - Older age;
  - Coexistence of carotid atherosclerosis;
  - Lower left ventricular ejection fraction; and
  - Smoking in patients with type 1 diabetes
(4) It seems that coexisting microvascular complications do not constitute an additional risk factor for the occurrence of silent heart ischemia.
(5) Assessments of both the occurrence and risk factors for silent heart ischemia in patients with long-term diabetes require further studies based on uniform diagnostic screening tests to assess the presence of coronary artery disease.

## Figures and Tables

**Table 1:** General characteristics of the studied group of patients (N = 104).

Data	In Total	Ischemia	No Ischemia	*p*-Value
	[N = 104]	[N = 24]	[N = 80]	[χ^2^/ANOVA]
Age [years]	48.0 ± 14.6	58.0 ± 11.6	45.0 ± 14.1	<0.001
Gender: female [N; %]	56; 53.8	10; 41.7	46; 57.5	0.17
Duration of diabetes [years]	19.6 ± 9.3	20.0 ± 11.3	19.5 ± 8.8	0.82
BMI [kg/m^2^]	26.9 ± 5.1	27.7 ± 4.8	26.7 ± 5.3	0.39
HbA1c [%]	8.0 ± 1.4	7.9 ± 1.6	8.0 ± 1.4	0.67
HbA1c [mmol/mol]	63.9 ± 8.2	62.8 ± 6.0	63.9 ± 8.2	0.67
Total cholesterol concentration [mg/dL]	196.6 ± 42.7	198.0 ± 38.7	196.2 ± 44.0	0.85
HDL cholesterol concentration [mg/dL]	63.5 ± 20.4	62.9 ± 20.7	63.7 ± 20.5	0.87
LDL cholesterol concentration [mg/dL]	110.5 ± 37.3	116.9 ± 34.0	108.5 ± 38.3	0.34
Triglyceride concentration [mg/dL]	122.7 ± 85.9	108.6 ± 42.1	126.9 ± 95.0	0.36
eGFR [mL/min/m^2^]	93.0 ± 26.1	89.9 ± 19.8	93.9 ± 27.7	0.51
Smoker [N; %]	18; 17.3	7; 29.2	11; 13.8	0.08
Arterial hypertension [N; %]	58; 55.8	16; 66.7	42; 52.5	0.22
Mean left ventricular ejection fraction (EF) [%]	55.9 ± 3.4	53.9 ± 3.7	56.5 ± 3.2	0.001
Carotid atherosclerosis [N; %]	51; 49.0	18; 75.0	33; 41.3	0.004

Data are presented as mean ± SD.

**Table 2:** Characteristics of the study groups were divided based on ischemic and non-ischemic conditions.

Data	In Total	Ischemia [A]		No Ischemia [B]		*p*-Value	
	[N = 104]	Type 1	Type 2	Type 1	Type 2	A	B
		[N = 12]	[N = 12]	[N = 55]	[N = 25]	[χ2/ANOVA]	
Age [years]	48.0 ± 14.6	52.0 ± 10.1	64.0 ± 10.0	39.1 ± 11.8	57.7 ± 9.7	0.01	<0.001
Gender: Women [N; %]	56; 53.8	5; 41.7	5; 41.7	36; 65.5	10; 40.0	1.00	0.03
Duration of diabetes [years]	19.6 ± 9.3	27.0 ± 11.1	15.2 ± 6.6	22.3 ± 7.7	13.7 ± 7.2	0.01	<0.001
BMI [kg/m^2^]	26.9 ± 5.1	27.0 ± 5.1	28.4 ± 4.5	24.7 ± 4.1	31.0 ± 5.2	0.49	<0.001
HbA1c [%]	8.0 ± 1.4	7.8 ± 1.0	8.0 ± 2.1	8.2 ± 1.4	7.7 ± 1.4	0.79	0.16
HbA1c [mmol/mol]	63.9 ± 8.2	61.7 ± 5.8	63.9 ± 12.3	66.1 ± 8.2	61 ± 8.2	0.79	0.16
Total cholesterol concentration [mg/dL]	196.6 ± 42.7	206.5 ± 46.8	189.5 ± 28.0	199.4 ± 44.7	189.1 ± 42.4	0.29	0.33
HDL cholesterol concentration [mg/dL]	63.5 ± 20.4	69.8 ± 22.9	56.0 ± 16.3	69.5 ± 15.6	51.0 ± 24.2	0.10	<0.001
LDL cholesterol concentration [mg/dL]	110.5 ± 37.3	117.4 ± 39.2	116.5 ± 29.7	110.8 ± 39.2	103.2 ± 36.2	0.95	0.43
Triglyceride concentration [mg/dL]	122.7 ± 85.9	97.6 ± 37.2	119.6 ± 45.4	100.5 ± 55.7	185.0 ± 132.8	0.21	<0.001
eGFR [mL/min/m^2^]	93.0 ± 26.1	94.3 ± 22.1	85.4 ± 17.1	99.0 ± 29.2	82.7 ± 20.4	0.28	0.01
Smoker [N; %]	18; 17.3	4; 33.3	3; 25.0	5; 9.1	6; 24.0	0.65	0.07
Arterial hypertension [N; %]	58; 55.8	6; 50.0	10; 83.3	20; 36.4	22; 88.0	0.08	<0.001

Data are presented as means ± SD.

**Table 3:** Multiple logistic regression results for factors influencing the incidence of silent ischemia.

	In Total	Type 1 Diabetes	Type 2 Diabetes
Age [10 years]	2.06 (1.38–3.08) ***	2.53 (1.34–4.77) **	2.08 (0.90–4.83)
Duration of diabetes [10 years]	1.06 (0.65–1.72)	1.36 (0.67–2.78)	1.48 (0.57–3.88)
BMI (5 kg/m^2^)	1.21 (0.79–1.85)	1.74 (0.88–3.44)	0.56 (0.25–1.25)
HbA1c [1%]	0.93 (0.67–1.30)	0.78 (0.46–1.33)	1.11 (0.73–1.69)
WHR [waist-hip ratio] [0.1]	1.51 (0.97–2.35)	1.87 (0.98–3.54)	0.50 (0.15–1.67)
Android obesity (+/−)	1.86 (0.74–4.67)	2.56 (0.63–10.33)	0.63 (0.14–2.86)
Hypertension (+/−)	1.81 (0.70–4.70)	1.75 (0.50–6.16)	0.68 (0.10–4.74)
GFR [10 mL/min/1.73 m^2^]	0.94 (0.78–1.13)	0.94 (0.74–1.19)	1.08 (0.75–1.57)
Smoker (+/−)	2.58 (0.87–7.65)	5.00 (1.10–22.68) *	1.06 (0.21–5.21)
Left ventricular ejection fraction EF% (1%)	1.27 (1.09–1.47) **	1.27 (1.03–1.54) *	1.25 (0.99–1.56)
Carotid atherosclerosis	4.09 (1.46–11.43) **	5.57 (1.45–21.42) *	1.58 (0.27–9.31)

* *p* < 0.05, ** *p* < 0.01, *** *p* < 0.001.

## Data Availability

Data available on request due to restrictions e.g., privacy or ethical. The data presented in this study are available on request from the corresponding author.
